# The Cheapest Disinfectant

**Published:** 1864-09

**Authors:** Robert Druitt

**Affiliations:** London


### THE CHEAPEST DISINFECTANT.

By Robert Druitt, Esq., London.

I ask permission to recommend to the readers of the *Medical Times and Gazette* a strong solution of iodine in methylated spirit, as a safe, cheap, and efficient disinfectant. Iodine is 8d. per ounce wholesale, and methylated spirit 4s. per gallon; therefore, supposing 4 oz. of iodine dissolved in a gallon of spirit cost 6s. 8d., allow a like sum for bottles, corks and profit, and we ought to get for 6d. about 6 oz. of the tincture.

The advantages of Iodine are (I believe it was first proposed as a disinfectant by Dr. Richardson) that it purifies solid surfaces as well as Condy's and Burnett's liquids; and that it is also volatile and acts on the air like chloride of lime, without its abominably nauseous odor.

In every house where there are sinks, closets, &c., internally, it is a good plan to disinfect them thoroughly from time to time in hot weather. Dust-bins should be treated with something to neutralize the emanations of the decaying vegetable rubbish thrown into them. When there is sickness in a house, and a *chaise percee* has to be used, it is very disgusting to have the contents taken without any precaution and allowed to waft a perfume all over the house. For all these purposes, for which a *liquid* disinfectant is convenient, I believe my professional brethren will find the tincture of iodine the most handy and effectual.

---

*Death from Chloroform.*—A young man named Denham Edward Saffery, the son of a chemist at Miletown, Sheerness, has recently died from the self-administration of chloroform, which he occasionally inhaled to relieve pain in the chest.—*Lancet, July* 18, 1864.

